# Nanogels Based on N,N-Dimethylacrylamide and β-Cyclodextrin Triacrylate for Enhanced Solubility and Therapeutic Efficacy of Aripiprazole

**DOI:** 10.3390/gels10040217

**Published:** 2024-03-22

**Authors:** Siyka Stoilova, Dilyana Georgieva, Rositsa Mihaylova, Petar D. Petrov, Bistra Kostova

**Affiliations:** 1 Department of Pharmaceutical Technology and Biopharmacy, Faculty of Pharmacy, Medical University of Sofia, Dunav Str. 2, 1000 Sofia, Bulgaria; s_stoilova@polymer.bas.bg (S.S.); dgeorgieva@pharmfac.mu-sofia.bg (D.G.); 2 Institute of Polymers, Bulgarian Academy of Sciences, Akad. G. Bonchev Str., Bl. 103-A, 1113 Sofia, Bulgaria; 3 Department of Pharmacology, Pharmacotherapy and Toxicology, Faculty of Pharmacy, Medical University of Sofia, 2 Dunav St., 1000 Sofia, Bulgaria; rmihaylova@pharmfac.mu-sofia.bg

**Keywords:** drug delivery systems, nanoparticles, nanogel, aripiprazole, antipsychotic drugs delivery

## Abstract

Aripiprazole (ARZ) is a medication used for the treatment of various diseases such as schizophrenia, bipolar disorder, major depressive disorder, autism, and Tourette’s syndrome. Despite its therapeutic benefits, ARZ is characterized by a poor water solubility which provoked the development of various delivery systems in order to enhance its solubility. In the present work, a nanoscale drug delivery system based on N,N-dimethylacrylamide (DMAA) and β-cyclodextrin triacrylate (β-CD-Ac_3_) as potential aripiprazole delivery vehicles was developed. The nanogels were synthesized by free radical polymerization of DMAA in the presence of β-CD-Ac_3_ as a crosslinking agent and then loaded with ARZ via host-guest inclusion complexation. The blank- and drug-loaded nanogels were evaluated using different methods. Fourier transform infrared (FTIR) spectroscopy was employed to confirm the incorporation of β-CD moieties into the polymer network. Dynamic light scattering (DLS) was used to study the size of the developed systems. The samples exhibited a monomodal particle size distribution and a relatively narrow dispersity index. The hydrodynamic diameter (D_h_) of the gels varied between 107 and 129 nm, with a tendency for slightly larger particles as the β-CD-Ac_3_ fraction increased. Loading the drug into the nanocarrier resulted in slightly larger particles than the blank gels, but their size was still in the nanoscopic range (166 to 169 nm). The release profiles in PBS were studied and a sustained release pattern with no significant burst effect was observed. A cytotoxicity assessment was also conducted to demonstrate the non-toxicity and biocompatibility of the studied polymers.

## 1. Introduction

Aripiprazole (ARZ), with chemical name 7-[4-[4-(2,3-dichlorophenyl)piperazin-1-yl]-butoxy]-3,4-dihydro-1H-quinolin-2-one, is an achiral quinolinone derivative [1]. It is a versatile medication utilized for the treatment of various diseases such as schizophrenia, bipolar disorder, major depressive disorder, autism, and Tourette’s syndrome [2]. ARZ possesses a specific pharmacological profile, acting as a partial agonist at the dopamine D_2_ and serotonin 5HT_1A_ receptors while antagonizing the serotonin 5HT_2A_ receptor. Notably, ARZ exhibits fewer side effects compared to other antipsychotic drugs, such as extrapyramidal syndrome, hyperprolactinemia, weight gain, metabolic disorders, and sedation [3].

Despite its therapeutic benefits, ARZ faces a solubility challenge, being poorly soluble in water (less than 0.3 mg L^−1^). According to the biopharmaceutical classification system, it belongs to the category of class IV drugs, characterized by low permeability and bioavailability. Consequently, significant efforts have been devoted to developing delivery technologies aimed at enhancing aripiprazole’s solubility and therapeutic efficacy.

Awais et al. have prepared binary and ternary inclusion complexes of ARZ with methyl-β-cyclodextrin (MβCD) and L-Arginine (LA), which successfully increased the drug’s dissolution rate [4]. Chennuri et al. have developed liquid and solid self-emulsifying drug delivery systems for aripiprazole [5]. Devangan et al. explored the incorporation of ARZ into mesoporous silica through a wet impregnation method [6]. Hedayati et al. introduced a novel poly(hydroxybutyrate-co-hydroxyvalerate) (PHBV)-based nanoporous in situ implant system for aripiprazole delivery [7]. Lin-Fei Chen et al. demonstrated the fabrication of aripiprazole and poly (methylvinylether-co-maleic anhydride) (PVMMA) composite nanoparticles (NPs) using the supercritical antisolvent (SAS) process to enhance water solubility and antidepressant effects [8]. Łyszczarza et al. contributed to the formulation process development of orodispersible films containing aripiprazole [9]. It is evident that polymeric carriers have emerged as promising materials for improving aripiprazole’s solubility and modulating its drug release rate, ultimately enhancing patient treatment efficacy [10,11,12,13,14,15,16,17].

Among various techniques employed to enhance solubility, the formation of inclusion complexes stands out as a precise method to improve the aqueous solubility, dissolution rate, and bioavailability of poorly water-soluble drugs [18]. Inclusion complexes between host–guest molecules can improve the aqueous solubility, increase the bioavailability and the physicochemical stability of the active molecules, and modify the drug delivery [19,20,21,22,23,24]. Natural and modified cyclic oligosaccharide cyclodextrins (CDs) can form host-guest complexes with suitable hydrophobic molecules due to their hydrophobic cavities [25]. CD inclusion complexes with bioactive compounds have led to extensive investigations in several different application areas aimed to overcome the limitations of certain substances [23,24,26,27,28]. Although CDs can be used alone, their combinations with different polymers can improve the characteristics of the drug carriers.

Poly-N,N-dimethylacrylamide (PDMAA) is a nonionic, hydrophilic, biocompatible polymer, well known for its remarkable water solubility and strong adhesion to various surfaces. Linear, crosslinked copolymers and blends based on PDMAA find a lot of applications in the medical and pharmaceutical sectors. Therefore, the development of novel PDMAA-based materials with modulated characteristics has gained increasing interest [29].

Recently, Danov et al. have described the preparation of a PDMAA/β-CD cryogel macrosystem for the delivery of ARZ [30]. The promising results obtained in this work motivated our team to develop PDMAA/β-CD nanogel carriers of ARZ.

Nanogels, a class of hydrogels ranging in size from approximately 10 nm to 200 nm, play a main role in drug delivery [31,32]. Nanogels can be obtained through both physical and chemical crosslinking mechanisms. In physical crosslinking, amphiphilic polymers self-assemble in water via non-covalent hydrophobic interactions. In contrast, chemical crosslinking involves covalent bond formation, yielding soft spherical 3D structures with a high water absorption capacity, ensuring shape retention, and preventing disintegration in liquids [33]. The three-dimensional hydrophilic networks of nanogels enable the encapsulation of drugs or biological macromolecules, while maintaining its native conformation [34]. Nanogel crosslinked structures impart a high stability of the carrier in harsh physiological environments [35]. Hence, nanogels have gained popularity as a versatile therapeutic delivery system due to their biocompatibility, ease of modification, stability, prolonged circulation which facilitates cellular uptake, and controlled drug release [36].

In this study, we report the development of novel PDMAA/β-CD nanogel carriers for ARZ. To the best of our knowledge, this is the first work describing a nanoscale drug delivery system for aripiprazole based on dimethylacrylamide and β-cyclodextrin triacrylate. Nanogels were obtained by the free radical polymerization of DMAA in the presence of a given amount of β-CD-Ac_3_ as a crosslinking agent. The effect of the mass ratio of reagents on the reaction efficiency and physico-chemical properties of the gels was evaluated by UV–Vis and FTIR spectroscopy, dynamic light scattering, and atomic force microscopy (AFM). Next, nanogels were loaded with ARZ, via host-guest inclusion complexation, and the drug-loading efficiency, drug release profile, and cytotoxicity were determined as well.

## 2. Results and Discussion

### 2.1. Synthesis of Nanogels

**Figure 1:**
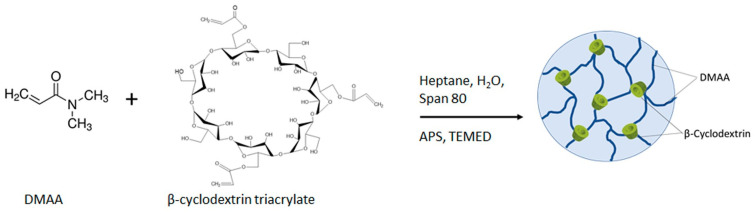
Synthetic scheme of preparation of the poly (N,N-dimethylacrylamide)/β-cyclodextrin triacrylate nanogel.

The nanogels were synthesized by free radical polymerization/crosslinking of N,N-dimethylacrylamide and β-CD-Ac_3_ in a hexane/water emulsion, using an ammonium persulfate (APS)/tetramethylethylenediamine (TEMED) redox system as an initiator and Span 80 as a surfactant (Figure 1). The emulsion consisted of aqueous compartments surrounded by a continuous organic phase. Since all reagents were water-soluble, the reaction proceeded in the dispersed aqueous phase, while Span 80 acted as a stabilizer. The polymer nanonetwork was formed under mild conditions by a well-known mechanism, involving an initiation step in which the primary radical species, generated from the reaction of APS with TEMED, triggers the polymerization of DMAA [37,38]. Next, in the propagation step, the linear macroradicals reacted with acrylate groups of the trifunctional β-CD-based crosslinking agent to form the nanogel network. β-CD was selected for its ability to form inclusion complexes with hydrophobic molecules, thereby enhancing their water solubility, dissolution rate, and bioavailability. The synthesis procedure of β-CD-based crosslinking agent, bearing three reactive acrylate groups, was described by us in a previous paper [30]. Briefly, hydroxyl groups of β-CD were reacted with acryloyl chloride in the presence of triethylamine. Given molar excess of acryloyl chloride was used to ensure attachment of three acrylate groups to each β-CD molecule. Nanogels of three different compositions were synthesized from DMAA and β-CD-Ac_3_, blended at molar ratio of 5:1, 2.5:1, and 1:1 (Table 1). For comparison, an additional nanogel based on DMAA and N,N′-methylenebisacrylamide (1:1 molar ratio), was synthesized as well. The calculated reaction yield (Table 1) revealed that with increasing β-CD-Ac_3_ fraction, the reaction efficiency decreased. This fact indicates a lower reactivity of the bulky crosslinking agent as compared to the monomer DMAA.

### 2.2. Fourier-Transform Infrared Spectroscopy

**Figure 2:**
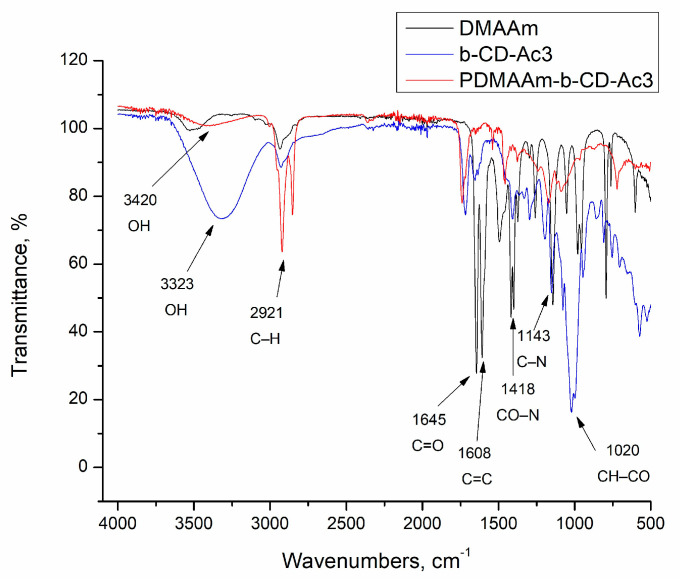
FTIR spectra of the two reagents, DMAA and β-CD-Ac_3_, and PDMAA-β-CD-Ac_3_ nanogel.

FTIR spectroscopy was employed to confirm the incorporation of β-CD moieties into the polymer network. The spectra of the reagents and the corresponding nanogel are shown in Figure 2.

The FTIR spectrum of DMAA has characteristic peaks at 1645 cm^−1^ for C=O stretching, 1608 cm^−1^ for C=C double bonds, and 1418 cm^−1^ for CO–N stretching [39,40]. In the FTIR spectrum of β-CD-Ac_3_, the band at 3323 cm^−1^ is assigned to the vibration of symmetrical and asymmetrical stretching of the –OH groups, and the peak at 1020 cm^−1^ is associated with the vibrations of the symmetric stretching CH–CO [41]. The peaks characteristic of C=O and C=C groups (1645 and 1608 cm^−1^) from the acrylate units are also detected. The presence of peaks related to C–H bonds (2921 cm^−1)^ and O–H groups (3420 cm^−1^) in the PDMAA:β-CD-Ac_3_ nanogel suggests that the polymerization of DMAA and β-CD-Ac_3_ was conducted successfully, and β-CD units were incorporated into the polymer network.

### 2.3. UV–Vis Spectroscopy

Next, the quantity of β-CD in the nanogels was determined by a method exploiting the ability of β-CD to form stoichiometric “host-guest” complexes with phenolphthalein (PhPh). PhPh dissolved in aqueous media is known to exhibit characteristic absorption with maxima at 554 nm, while the inclusion of PhPh into the β-CD cavity decreases the absorption proportionally to the fraction of the dye involved in the complex [42].

**Figure 3:**
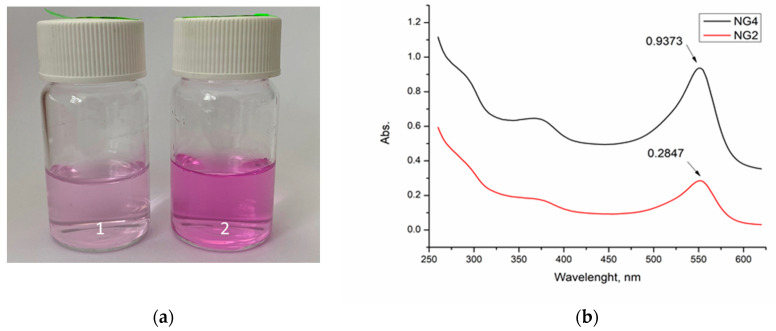
(**a**) Digital images of nanogels synthesized from DMAA and β-CD-Ac_3_ (1, NG2) and BAA (2, NG4), and (**b**) UV–Vis spectra of nanogels synthesized from DMAA and β-CD-Ac_3_ (NG2) and BAA (NG4).

Our initial dissolution experiments with the PhPh-based samples indicated that the loading capacity of the nanogels depended on their composition. In fact, the purple color of the nanogel solutions was less intense for the gels synthesized with a larger fraction of β-CD-Ac_3_. In contrast, mixing PhPh with the nanogel obtained with BAA as the crosslinker (the control sample) did not change the color/absorbance of the solution (Figure 3). The color change suggests that PhPh molecules were embedded in the nanogels comprising β-CD units; the gel without β-CD did not preferably incorporate dye molecules into the network. The precise calculation of β-CD content in the nanogels was done with the aid of UV–Vis spectroscopy, using a calibration curve, and the results are given in Table 1. It is obvious that the higher the initial fraction of β-CD-Ac_3_ in the reaction mixture, the higher the content of β-CD in the polymer network. In addition, these tests confirmed our suggestion that β-CD-Ac_3_ was less reactive than DMAA. This fact can be attributed to the bulkier molecule of the crosslinking reagent and its relatively lower concentration in the initial reaction mixture as compared to DMAA.

### 2.4. Drug Loading

The affinity of aripiprazole for complexation with β-CD is due to the hydrophobic cavity in the oligosaccharide structure. This phenomenon was considered to play a key role in the process of ARZ loading into the nanogels. Considering the composition and physico-chemical properties of the developed nanocarriers, we selected the nanogels comprising relatively large number of β-CD units within the polymer network (NG1 and NG2) for further experiments on drug loading and release. The results of the loading of aripiprazole in the systems showed that the loading efficiency (LE) was not significantly affected by the content of β-CD-Ac_3_ in the nanogel. LE of samples NG1and NG2 were 36.8% and 38.5%, respectively.

### 2.5. Dynamic Light Scattering and ζ-Potential Measurements

**Figure 4:**
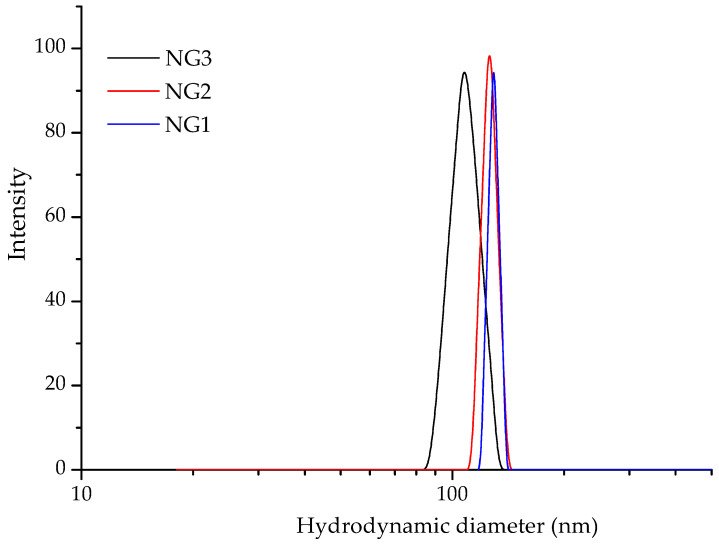
Hydrodynamic diameter distribution plots of blank nanogels comprising different number of β-CD units.

Some important physico-chemical characteristics of the nanogels were determined by using DLS and electrophoretic light scattering (ELS) analyses. Table 1 lists the hydrodynamic diameter, dispersity index, and ζ-potential of the gels as a function of their composition. The samples exhibited a monomodal particle size distribution and a relatively narrow dispersity index (Figure 4). The hydrodynamic diameter of the gels varied between 107 and 129 nm, with a tendency for slightly larger particles as the β-CD-Ac_3_ fraction increased. The surface charge of the nanogels was negative, which was considered advantageous for providing superior colloid stability of the systems. The negative ζ-potential values were probably due to specific interactions involving the coordination of hydroxyl anions with amino groups present on the surface of the nanogel particles. The nanogel systems remained stable upon storage at room temperature for 4 weeks and no precipitation or significant changes of D_h_ were observed. Therefore, we can conclude that the developed carriers are suitable for different types of drug administration.

DLS analysis provides valuable insights into how the loading of aripiprazole affects the nanogels in terms of size and surface charge, which are considered crucial factors for their performance and potential applications in drug delivery and related fields. ARZ-loaded nanogel systems maintained a low dispersity index, indicating a narrow particle size distribution. Embedding the drug into the nanocarrier resulted in slightly larger particles than the blank gels, but their size was still in the nanoscopic range (Table 1). The ζ-potential values decreased after aripiprazole loading from −18.3 mV to −8.7 mV for NG1 and from −21.8 mV to −7.2 mV for NG2. This slight change suggests an interaction between the drug molecules and the carriers, leading to partial removal of the coordinated anions from the carrier surface. However, the good colloidal stability of the systems was not affected.

### 2.6. Atomic Force Microscopy (AFM)

**Figure 5:**
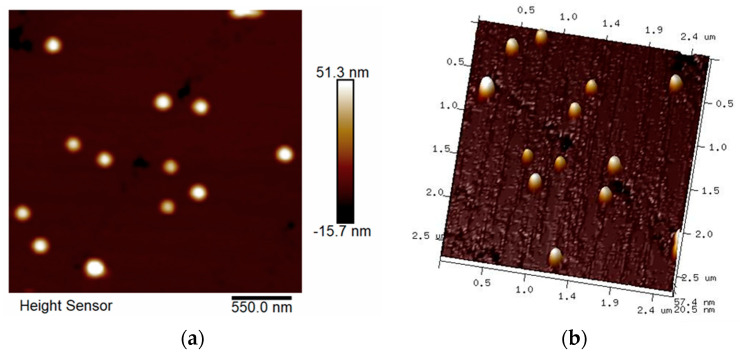
Representative AFM 2D (**a**) and 3D (**b**) height images of nanogel synthesized from DMAA and β-CD-Ac_3_ at 1:1 feed ratio (NG1).

Atomic force microscopy analysis was used to assess the morphology of the nanogels. Representative micrographs of NG1 are shown in Figure 5. As can be seen from the images, the particles were spherical in shape, and possessed an average diameter of 187 ± 4 nm. It should be mentioned that AFM images were obtained by spin-coated nanogel solutions and, typical of such procedures, the particles can be slightly flattened. Nevertheless, the results from AFM were consistent with the DLS data.

### 2.7. Drug Release Studies

**Figure 6:**
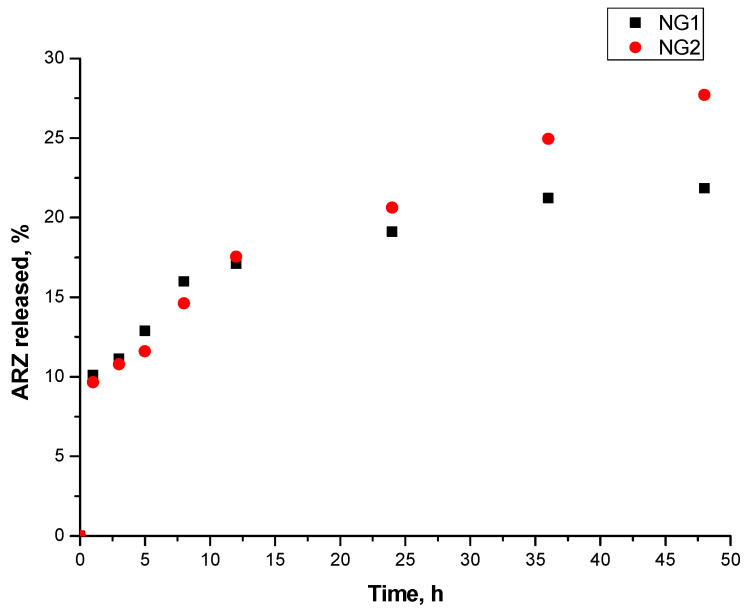
In vitro release of aripiprazole from the two nanogel samples.

The in vitro release tests (Figure 6) revealed that the release profiles have a relatively consistent rate, without significant burst effects. Thus, a sustained release pattern is evident, reaching 21.82% and 27.72% of released drug within 48 h for NG1 and NG2, respectively. These low values are most likely due to the very low solubility of ARZ, which is a weak basic drug and exhibits pH-dependent solubility [17,43]. It is most soluble at an acidic pH and has a very low solubility at a pH above six (at which the release study was conducted).

In the initial stage of the release process, the two formulations displayed a similar release pattern, likely attributed to the release of the superficially located ARZ and the higher concentration gradient. A slight difference in the amount of the released ARZ can be noticed after 24 h, with the NG2 formulation exhibiting a more pronounced drug release. This difference can be attributed to the higher amount of DMAA which imparts hydrophilicity to the structure.

This observation emphasizes the potential benefits of formulating compositions with different β-CD-Ac3 contents, which can effectively enhance the efficiency of the system and optimize the drug release profile for improved therapeutic results.

### 2.8. Cytotoxicity Assessment

**Figure 7:**
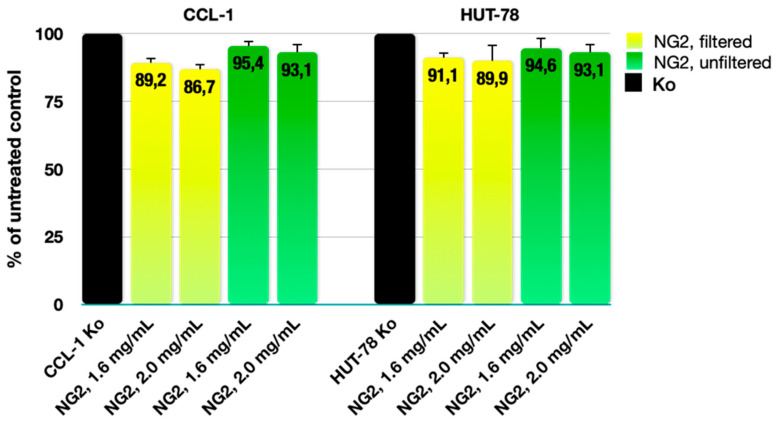
Effect of filtered and unfiltered NG2 nanogel on the cell viability in a normal murine fibroblast cell line (CCL-1) and a cutaneous T-cell lymphoma in vitro model (HUT-78) after continuous 72 h exposure to two different concentrations.

The biocompatibility of any polymeric materials is a prerequisite for their potential application as transdermal drug delivery systems. Accordingly, we performed a series of MTT experiments with CCL-1 and HUT-78 cell lines to rule out any cytotoxic behavior of both filtered and unfiltered NG2 samples, in two treatment concentrations (2.0 mg/mL and 1.6 mg/mL). The cell viability data (Figure 7) in both cell lines are coherent and the interference in cell growth was negligible across all treated samples (less than 15%), compared to the untreated control. In the CCL-1 fibroblast cells, the variations in the inhibitory effect, produced by equivalent doses of the filtered and unfiltered nanogel, amount to merely 6–7%, and were twice as small in the case of the lymphoblastic HUT-78 cell line. Both filtered and unfiltered material induced no morphological changes under light microscopic examination and no subsequent changes were detected in cell appearance regarding size, shape, membrane integrity, detachment, and cell-cell contacts.

Based on the results obtained in the cell viability study, the tested NG2 nanogel can be considered non-toxic and biocompatible even at concentrations as high as 2.0 mg/mL, far exceeding the potential systemic exposure in its intended application.

## 3. Conclusions

In the present work, an original nanoscale drug delivery system based on dimethylacrylamide and β-cyclodextrin triacrylate was developed for potential transdermal aripiprazole delivery. The nanogels were first obtained by free radical polymerization of DMAA in the presence of β-CD-Ac_3_ as a crosslinking agent and then loaded with ARZ via host-guest inclusion complexation. FTIR spectroscopy proved that the polymerization of DMAA and β-CD-Ac_3_ was conducted successfully, and β-CD units were incorporated into the polymer network. The quantity of β-CD in the nanogels was determined using a method exploiting the ability of β-CD to form stoichiometric “host-guest” complexes with phenolphthalein. The precise calculation of the β-CD content in the nanogels was made with the aid of UV–Vis spectroscopy. It was found that the higher the initial fraction of β-CD-Ac_3_ in the reaction mixture, the higher the content of β-CD in the polymer network. In addition, these tests confirmed that β-CD-Ac_3_ was less reactive than DMAA.

The nanosized structure of the systems was observed by AFM and confirmed by DLS. The samples exhibited monomodal particle size distributions and a relatively narrow dispersity index. The D_h_ of the blank gels varied between 107 and 129 nm, with a tendency for slightly larger particles as the β-CD-Ac_3_ fraction increased. Loading the drug into the nanocarrier resulted in slightly larger particles than the blank gels, but their size was still in the nanoscopic range (166 to 169 nm).

The release profile tests in PBS at 37 °C revealed a sustained release pattern with no significant burst effect. In the initial stages of the release process, the different formulations displayed a similar release pattern, likely attributed to the release of the superficially located aripiprazole. However, a noticeable deviation occurred after 24 h, with the NG2 formulation exhibiting a more pronounced release of ARZ. This difference can be attributed to the higher hydrophilicity of the structure.

The conducted cytotoxicity assessment demonstrated that the developed nanogels are non-toxic and biocompatible, and can be considered suitable for delivery of ARZ.

## 4. Materials and Methods

### 4.1. Materials

DMAA, β-CD, acryloyl chloride, triethylamine (TEA), ammonium persulfate (APS), tetramethylethylenediamine (TEMED), hexane, and Span 80 were purchased from Sigma–Aldrich (FOT, Sofia, Bulgaria) and used as received. Aripiprazole was purchased from Fengchengroup (Qingdao, China). β-CD-Ac_3_ was synthesized as described elsewhere [30].

### 4.2. Methods

#### 4.2.1. Synthesis of Nanogels

Hexane (6.8 mL) and Span 80 (0.2 mL) were added in a round-bottom glass flask and homogenized with a magnetic stirrer at room temperature. Next, given amounts of DMAA and β-CD-Ac_3_ (1:1; 2.5:1; and 5:1 mass ratios, respectively, of 0.06:0.06 g; 0.08573:0.03 g; 0.1:0.02 g), 0.01 g of ammonium persulfate (APS), and 25 µL of tetramethylethylenediamine (TEMED) were dissolved in 0.2 mL of water and were added to the organic medium. After blending, the reaction mixture was purged with argon for 15 min to remove the oxygen. The free radical polymerization/crosslinking reaction was carried out overnight at a constant temperature of 30 °C under continuous stirring.

The purification procedure involved: the removal of hexane from the system using a vacuum evaporator; an extraction of the surfactant (Span 80) with methylene chloride; and dialysis of the aqueous phase, containing nanogel, against water for 5 days, utilizing a membrane (MWCO 12,000; Spectrum Labs, New Brunswick, NJ, USA). Finally, the nanogel was collected by lyophilization for 24 h.

#### 4.2.2. Fourier-Transform Infrared Spectroscopy

FTIR spectra of freeze-dried nanogels were recorded with an attenuated total reflection (ATR) spectrometer (IRAffinity-1, Shimadzu, Kyoto, Japan) in the 500 to 4000 cm^−1^ range.

#### 4.2.3. UV–Vis Spectroscopy

The quantitative fraction of β-CD-Ac_3_ in the nanogel was determined with the aid of phenolphthalein using UV–Vis spectroscopy. UV–Vis spectroscopy measurements were performed on a DU800 spectrophotometer (Beckman Coulter Inc., Brea, CA, USA). In the first step, a series of phenolphthalein complexed in water with a β-CD molar ratio from 0.1679 to 10.7 was prepared. The absorbance of phenolphthalein at 550 nm was recorded and then a calibration curve was plotted where y = 0.00808 + 34.7638x, with a correlation coefficient of R = 0.996. Next, a phenolphthalein solution with a concentration of 0.04 mg/mL was prepared. The nanogels were dissolved in water and solutions with a concentration of 0.6527 mg/mL for NG1 and 0.5219 mg/mL for NG2 were obtained. Each nanogel was added to the phenolphthalein solution by drip, mixed by stirring, and subjected to analysis.

#### 4.2.4. Drug Loading

Firstly, aripiprazole was dissolved in methanol. The methanolic solution of the drug was then slowly added to an aqueous solution of the nanogels. Two nanosystems were obtained: (i) by blending 7 mL of an aqueous solution of NG1 (2.857 mg/mL) with 7 mL of a methanolic solution of ARZ (0.7 mg/mL), and (ii) by mixing 7 mL of an aqueous solution of NG2 (2.857 mg/mL) with 7 mL of a methanolic solution of ARZ (0.7 mg/mL). The expected or theoretical content of aripiprazole in each system was 5 mg.

The loading efficiency (LE, %) was calculated according to the following equation:

LE = (A − B)/A × 100,

where A is the total amount of ARZ and B is the amount of free ARZ.

#### 4.2.5. Dynamic Light Scattering and ζ-Potential Measurements

The hydrodynamic diameter of the nanogels was determined with a Zetasizer NanoBrook 90Plus PALS instrument (Brookhaven Instruments Corporation, Holtsville, NY, USA), equipped with a 35-mW red diode laser (λ = 640 nm), at a scattering angle of 90°. The zeta potential was determined by the phase analysis light scattering (PALS) method at a scattering angle of 15°. The sample concentration was 1.0 g L^−1^ and five measurements were performed for each sample at a temperature of 25 °C.

#### 4.2.6. Atomic Force Microscopy

Atomic force microscopy analyses were carried out with a Bruker Dimension Icon microscope (Bruker Nano GmbH, Karlsruhe, Germany) under ambient conditions at a 1.00 Hz scan rate. 2 μL of the nanogel solution (1 g L^−^^1^) were dropped onto a freshly cleaned glass substrate and spin-coated at 2000 rpm. The images were taken in tapping mode, using silicon cantilevers with resonance frequency of 300 kHz and a spring constant of 40 N/m.

#### 4.2.7. Drug Release Studies

To assess the release profiles of ARZ from the nanogels, a water shaking bath (IKASH-B 20, Staufen, Germany) was employed. The drug-loaded nanogel solutions were carefully placed within dialysis membranes with a molecular weight cutoff of 12,000 Daltons and submerged in 100 mL of phosphate buffer solution (pH 6.8). These tests were conducted at an agitation speed of 50 rpm and a constant temperature of 37 ± 0.5 °C. At predefined time intervals, 2 mL samples were extracted for subsequent analysis. Following each sampling, the volume was replenished with an equivalent quantity of fresh, clean medium. The concentration of aripiprazole in the sample solution was determined using UV spectroscopy with absorbance measurements taken at 220 nm (Thermo Scientific Evolution 300 UV–Vis Spectrophotometer, Thermo Fisher Scientific, Lenexa, KS, USA).

#### 4.2.8. Cytotoxicity Assessment

##### Cell Lines and Culture Conditions

The in vitro cytotoxicity and biocompatibility of the tested materials were assessed against both normal murine fibroblast cells (CCL-1), as well as malignant human cutaneous T-cell lymphoma cells (HUT-78). Both cell lines were purchased from the German Collection of Microorganisms and Cell Cultures (DSMZ GmbH, Braunschweig, Germany) and cell cultures were maintained and grown in the appropriate culture medium specified by the provider (EMEM supplemented with 10% horse serum for the adherent CCL-1 cells, and IMDM, adjusted to a final 20% concentration of fetal bovine serum for the suspension HUT-78 cell line). Cell cultures were incubated under standard conditions of 37 °C and a 5% humidified CO_2_ atmosphere.

##### In Vitro MTT Colorimetric Assay

The inhibitory effect of the polymeric material on cell growth was evaluated using a validated methodology for assessing cell viability known as the Mosmann MTT method. Exponential-phased cells were harvested and seeded (100 μL/well) in 96-well plates at the appropriate density (3 × 105 for the suspension culture HUT-78 and 1.5 × 105 for the adherent CCL-1 cells). Following a 24 h incubation, cells were exposed to two different doses of both filtered and unfiltered polymer, equivalent to a 2 mg/mL and 1.6 mg/mL final concentration in the well. After an exposure time of 72 h, filter-sterilized MTT substrate solution (5 mg/mL in PBS) was added to each well of the culture plate. A further 1–4-h incubation allowed for the formation of purple insoluble formazan crystals. The latter were dissolved in isopropyl alcohol solution containing 5% formic acid prior to absorbance measurement at 550 nm using a microplate reader (Labexim LMR-1). Collected absorbance values were blanked against MTT and an isopropanol solution, and normalized to the mean value of untreated control (100% cell viability).

## Figures and Tables

**Table 1:** Composition, size, dispersity index, ζ-potential, and loading efficiency of the developed systems.

Sample Code	Composition DMAA/β-CD-Ac_3_ (BAA) Feed Ratio Calculated		Yield %	D_h_ nm	Dispersity Index	ζ-Potential mV	LE %
NG1	1:1	1.86:1	72 ± 2	129 ± 2	0.16 ± 0.03	−18.3 ± 0.5	-
NG2	2.5:1	3.15:1	73 ± 2	125 ± 2	0.16 ± 0.04	−21.8 ± 0.6	-
NG3	5:1	7.11:1	94 ± 3	107 ± 2	0.24 ± 0.04	−23.1 ± 0.6	-
NG4	1:1 *	n.a. **	85 ± 2	110 ± 2	0.17 ± 0.03	−8.5 ± 0.9	-
NG1-ARZ	1:1	1.86:1	-	169 ± 2	0.14 ± 0.02	−8.7 ± 0.8	36.8
NG2-ARZ	2.5:1	3.15:1	-	166 ± 2	0.13 ± 0.02	−7.2 ± 0.8	38.5

* BAA; ** n.a.: not applicable.

## Data Availability

The data presented in this study are openly available in article.
